# Different signalling in infarcted and non‐infarcted areas of rat failing hearts: A role of necroptosis and inflammation

**DOI:** 10.1111/jcmm.14536

**Published:** 2019-07-21

**Authors:** Martin Lichý, Adrián Szobi, Jaroslav Hrdlička, Csaba Horváth, Veronika Kormanová, Tomáš Rajtík, Jan Neckář, František Kolář, Adriana Adameová

**Affiliations:** ^1^ Faculty of Pharmacy, Department of Pharmacology and Toxicology Comenius University in Bratislava Bratislava Slovak Republic; ^2^ Institute of Physiology of the Czech Academy of Sciences Prague Czech Republic

**Keywords:** heart failure, inflammation, MLKL, necroptosis, RIP3

## Abstract

Necroptosis has been recognized in heart failure (HF). In this study, we investigated detailed necroptotic signalling in infarcted and non‐infarcted areas separately and its mechanistic link with main features of HF. Post‐infarction HF in rats was induced by left coronary occlusion (60 minutes) followed by 42‐day reperfusion. Heart function was assessed echocardiographically. Molecular signalling and proposed mechanisms (oxidative stress, collagen deposition and inflammation) were investigated in whole hearts and in subcellular fractions when appropriate. In post‐infarction failing hearts, TNF and pSer229‐RIP3 levels were comparably increased in both infarcted and non‐infarcted areas. Its cytotoxic downstream molecule p‐MLKL, indicating necroptosis execution, was detected in infarcted area. In non‐infarcted area, despite increased pSer229‐RIP3, p‐MLKL was present in neither whole cells nor the cell membrane known to be associated with necroptosis execution. Likewise, increased membrane lipoperoxidation and NOX2 levels unlikely promoted pro‐necroptotic environment in non‐infarcted area. Collagen deposition and the inflammatory csp‐1‐IL‐1β axis were active in both areas of failing hearts, while being more pronounced in infarcted tissue. Although apoptotic proteins were differently expressed in infarcted and non‐infarcted tissue, apoptosis was found to play an insignificant role. p‐MLKL‐driven necroptosis and inflammation while inflammation only (without necroptotic cell death) seem to underlie fibrotic healing and progressive injury in infarcted and non‐infarcted areas of failing hearts, respectively. Upregulation of pSer229‐RIP3 in both HF areas suggests that this kinase, associated with both necroptosis and inflammation, is likely to play a dual role in HF progression.

## 1 INTRODUCTION

Because adult cardiomyocytes are terminally differentiated and have a very low rate of cell cycle re‐entry and proliferation,^1^ the heart possesses a very limited capacity to regenerate. This feature is important in particular for conditions characterized by a loss of the functional myocardium such as myocardial infarction (MI) which can progress to heart failure (HF). Although excessive research has been undertaken to investigate certain cell death modalities in such damaged heart, it is still unclear which of them underlie its phenotypes. Recent studies investigating newly recognized cell death modalities have suggested the possible involvement of necroptosis in the pathogenesis of post‐MI HF.^2^, ^3^, ^4^, ^5^ The presence and interaction of the main necroptotic proteins have been detected in advanced human HF,^5^ while both genetic knock‐out and pharmacological inhibition of necroptotic signalling has been shown to alleviate deleterious cardiac phenotypes in HF, including contractile dysfunction, remodelling and inflammation.^2^, ^3^, ^6^

Signalling of necroptosis, a form of regulated cell death resembling morphological features of necrosis^7^, ^8^, ^9^, ^10^ proceeds due to a formation of the necrosome complex containing phosphorylated protein kinases RIP1 and RIP3 to further phosphorylate MLKL, a terminal pro‐necroptotic protein.^11^ As a result, such phosphorylated MLKL molecules at Thr357 and/or Ser358 oligomerize and translocate into the plasma membrane causing the execution of the necrotic‐like cell death by inducing alterations in ion homoeostasis and plasma membrane disruption.^12^, ^13^, ^14^, ^15^ Recently, it has been suggested that RIP3 and MLKL activation can also be associated with inflammation via the activation of NLRP3 inflammasome and IL‐1β independently on the necroptotic cell loss.^16^, ^17^, ^18^

In view of the fact that post‐MI HF is positive for main necroptotic proteins,^2^, ^5^, ^6^ we tested a hypothesis that necroptosis is responsible for cell loss of the infarcted myocardium. Likewise, we hypothesized that necroptosis in the infarcted area, due to disruption of the plasma membrane leading to the release of intracellular content and pro‐inflammatory mediators, causes diffuse pro‐inflammatory and/or pro‐necroptotic injury of the surrounding non‐infarcted tissue. These cellular events can further promote fibrotic healing of the injured zone, as well as interstitial fibrosis of the non‐infarcted tissue with subsequent changes in left ventricle geometry, and cardiac remodeling. In addition, a disproportionate accumulation of the released inflammatory mediators can contribute to the progressive impairment of both cardiac contraction and relaxation and thereby underlie other phenotypes of HF.

## 2 MATERIALS AND METHODS

### 2.1 Animal model and study design

All procedures conform to the Guide for the Care and Use of Laboratory Animals published by the US National Institutes of Health (NIH publication No. 85‐23, revised 1996) and have been authorized by the Animal Care and Use Committee of the Institute of Physiology of the Czech Academy of Sciences (No. 76/2016).

**Figure 1:**
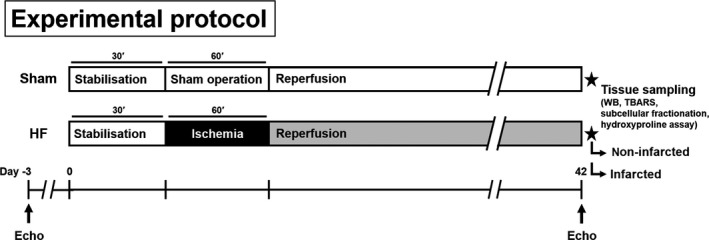
Illustration showing experimental protocol.; Sham—group subjected to surgery without coronary occlusion; HF—group subjected to 60 min coronary occlusion with subsequent development of HF over the course of 42 days

**Figure 2:**
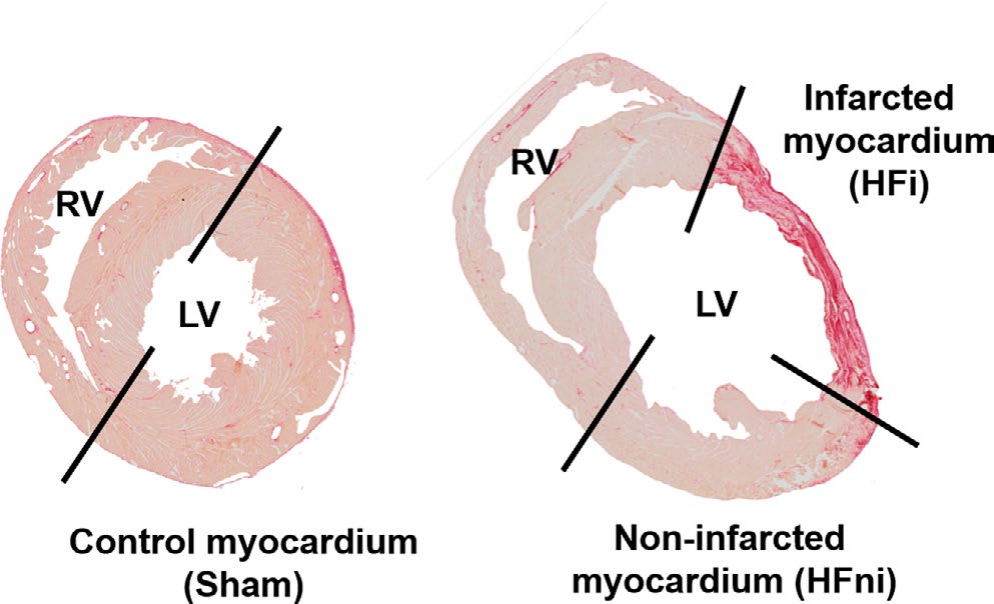
Example of transverse sections of sham‐operated (left) and failing (right) hearts stained with Picrosirius Red for a delineation of infarcted and non‐infarcted area of the left ventricle. Black lines illustrate place of dissection

Adult male Hannover Sprague‐Dawley rats (250‐300 g, IKEM, Prague, Czech Republic) housed in a room with constant temperature of 22°C, and 12h:12h light/dark cycle were fed with a standard pellet diet and tap water ad libitum. After incubation period, rats were randomly assigned into two groups (Figure 1): sham‐operated animals (Sham, n = 9) and group subjected to MI with subsequent development of HF (n = 10).^19^ In anesthetized open‐chest animals (sodium pentobarbital, 60 mg/kg *i.p*.), MI was induced by ligation of the left coronary artery 1‐2 mm distal to the left atrial appendage for 1 hour. After this period, ligation was released. Sham‐operated rats underwent chest surgery without occlusion. After chest closure, all spontaneously breathing animals recovering from anaesthesia were housed in separate cages with given analgesia (ibuprofen, 20 mg/day *p.o*.) for another 3 days. Mortality of rats with MI was 43%. Echocardiography was performed 3 days before and 42 days after the surgical procedure by using GE Vingmed System Five (GE Vingmed Ultrasound) and FPA 10 MHz probe (GE Vingmed Ultrasound). Animals were anesthetized with 2% isoflurane (Forane; Abbott Laboratories) mixed with room air. Left ventricular (LV) systolic (LVDs) and diastolic diameters (LVDd) were directly measured, from which fractional shortening (FS) was derived according to the formula FS (%) = [100 × (LVDd‐LVDs)/(LVDd)]. At the end of experiment the animals were sacrificed by pentobarbital overdose; blood samples were taken from the right ventricular cavity and hearts rapidly excised and washed in ice‐cold PBS. Whole free LV wall of sham‐operated animals was harvested while in the HF group, the LV was dissected into infarcted area (HFi) and remaining non‐infarcted tissue (HFni) (Figure 2) and stored at −80°C till further molecular and cellular analyses (Figure 1).

### 2.2 Hydroxyproline assay

Assay according to da Silva et al^20^ with minor adjustments was used to determine myocardial collagen content. Heart tissue was homogenized in phosphate buffer (20 mmol/L, pH = 8.0) with 1% SDS and the resulting suspension was transferred into two screw‐cap microtubes (duplicates). Afterwards, NaOH was added to a final concentration of 3.5 M. Samples were then incubated for 1 hour at 115°C to hydrolyze all proteins. After cooling, excess NaOH was neutralized with H_2_SO_4_, samples were centrifuged at 13 000 g for 10 minutes and the supernatant was used to determine hydroxyproline (Hyp) concentration. Samples were first mixed with 3 parts of Chloramine‐T reagent (1.6 M phosphate buffer pH = 6.0, 55 mmol/L Chloramine‐T, 10% isopropanol) and left to incubate for 25 minutes. Next, 3 parts of DMAB (p‐dimethylaminobenzaldehyde) reagent (1.2 M DMAB, 40% HClO_4_, 60% isopropanol) were added and the mixture was left to incubate at 75°C for 20 minutes. Finally, samples were spectrophotometrically measured at 560 nm. Collagen content was derived from Hyp concentration by dividing by 0.135 and expressed as a percentage of wet tissue weight.

### 2.3 Western blotting

Left ventricular tissue samples were processed for immunoblotting analysis by SDS‐PAGE and Western Blotting as described previously.^5^ Post‐electrophoresis, proteins were transferred onto PVDF membranes (Immobilon‐P, Merck Millipore) and incubated with primary antibodies against Bax (ab182734, Abcam), Bcl‐2 (SAB4500003, Sigma‐Aldrich), Caspase‐1 (ab179515, Abcam), Caspase‐3 (#9662, Cell Signaling Technology), Caspase‐7 (#12827, Cell Signaling Technology), Caspase‐8 (#4790, Cell Signaling Technology), GAPDH‐HRP (60004, Proteintech), IL‐1β (ab9722, Abcam), MLKL (MABC604, Merck Millipore), Na/K‐ATPase (#3010, Cell Signaling Technology), NOX2 (OABB00620, Aviva Systems Biology), Cleaved PARP‐1 p25 (ab32064, Abcam), RIP1 (#3493, Cell Signaling Technology; 610459, BD Bioscience), RIP3 (ARP32835_P050, Aviva Systems Biology), pSer229‐RIP3 (ab195117, Abcam) and TNF (NBP2‐45333, Novus Biologicals). Subsequently, membranes were incubated with HRP‐conjugated secondary antibodies: donkey anti‐rabbit IgG (711‐035‐152, Jackson Immunoresearch), donkey anti‐mouse IgG (115‐035‐174, Jackson Immunoresearch), donkey anti‐rat IgG (112‐035‐175, Jackson Immunoresearch) or bovine anti‐goat IgG (205‐032‐176, Jackson Immunoresearch). Signals were detected using enhanced chemiluminiscence (Crescendo Luminata, Merck Millipore) and captured by a chemiluminiscence imaging system (myECL imager, Thermo Scientific). Total protein staining of membranes with Ponceau S or Cu‐phthalocyanine sulphonate assessed by scanning densitometry was used as the loading control in total tissue lysates and subcellular fractions, respectively.^21^ Relative expression of protein bands of interest was calculated by normalizing the intensity of a protein band with its whole lane protein staining intensity. Immunoblotting analysis was performed separately for comparison of HFni versus HFi and Sham versus HFni, and expression of the particular protein was adjusted to its 100% reference expression in HFni.

### 2.4 Phos‐tag™ SDS‐PAGE

Phos‐tag™ acrylamide (Wako Pure Chemical Industries, Ltd.) was used for detection of mobility shift of phosphorylated proteins by SDS‐PAGE.^22^, ^23^ Briefly, Phos‐tag™ acrylamide (60 μmol/L) and ZnCl_2_ (120 μmol/L) were added to a standard gel solution, which was polymerized by addition of ammonium persulfate and TEMED. To neutralize the effect of divalent metal chelators in the lysates used for Phos‐tag™ SDS‐PAGE, these were supplemented with ZnCl_2_ at a concentration equal to concentrations of previously added chelating agents. All subsequent procedures including electrophoresis, transfer and immunodetection were carried out by following standard protocol for Western blotting as described above.

### 2.5 Subcellular fractionation

All procedures were performed on ice with pre‐chilled instruments, reagents and centrifuge (4°C) following a protocol described in detail in Szobi et al.^24^ Briefly, after homogenization of tissues in imidazole buffer (pH = 7.6) containing 600 mmol/L sucrose, the suspension was centrifuged at 800 g for 20 minutes. The supernatant was centrifuged at 800 g for 20 minutes first, then at 10 000 g for an additional 10 minutes. Finally, it was enriched with CaCl_2_ to achieve a concentration of ~10 mmol/L and centrifuged at 20 000 g for 20 minutes. The remaining pellet referring to the membrane fraction was re‐suspended in buffer containing detergents and EDTA/EGTA and incubated for 60 minutes while the supernatant representing the cytoplasmic fraction was further processed with detergents according to the above described Western blotting protocol.

### 2.6 Measurement of thiobarbituric acid reactive substances

For the measurement of lipid peroxidation in total lysates and membrane subcellular fraction, thiobarbituric acid reactive substances (TBARS) generation was evaluated. Homogenates were firstly precipitated with trichloroacetic acid and then thiobarbituric acid (TBA) was added. Samples were incubated at 95°C for 70 minutes and chilled subsequently. Precipitated products were centrifuged and discarded. The supernatant containing the pink malondialdehyde (MDA)‐TBA adduct was analysed spectrophotometrically at 530‐540 nm and expressed as content of MDA per total protein in the measured sample.

### 2.7 Statistical analysis

The results are expressed as means ± standard error of means (SEM). Two‐tailed unpaired Student's t test with or without Welch's correction was used for evaluation of differences between two groups while one‐way ANOVA analysis with Tukey's post hoc tests was used for evaluation of group differences in variables with normal distribution between three groups. In case of non‐normally distributed data, Mann‐Whitney *U* test was used instead. All analyses were performed with GraphPad Prism 7.00 for Windows (GraphPad Software). Differences between groups were considered significant when *P* < 0.05.

## 3 RESULTS

### 3.1 Heart function and myocardial remodeling

**Figure 3:**
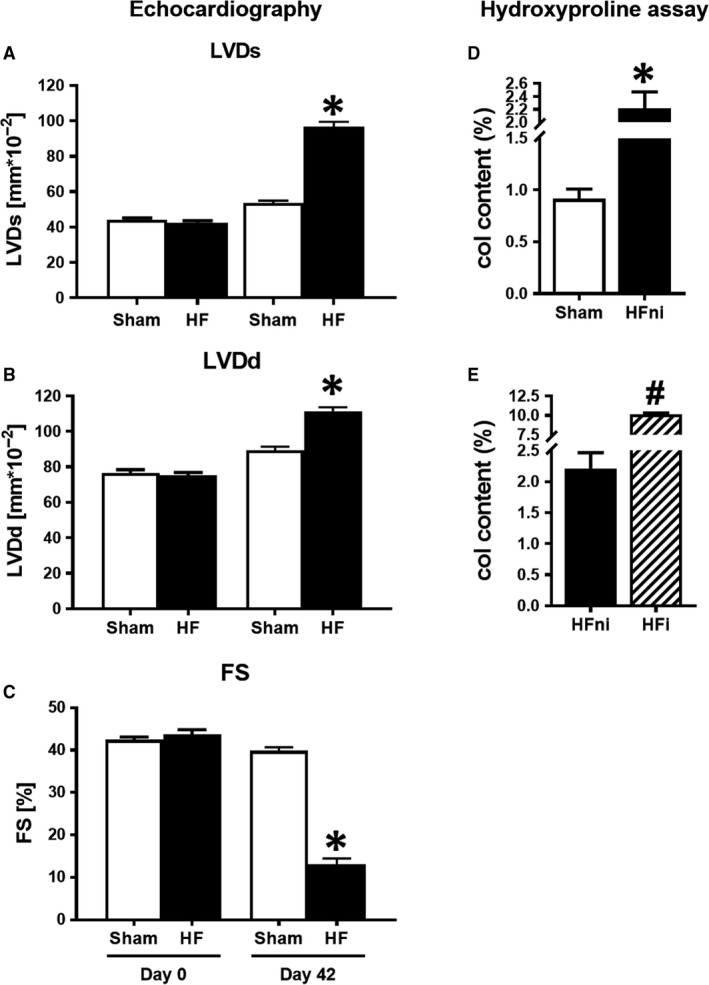
Left ventricular (LV) dilatation and contractile dysfunction. A‐C, Echocardiographic measurement of A, LVDs—LV systolic diameter; B, LVDd—LV diastolic diameter; and C, FS—fractional shortening of sham‐operated (Sham) and failing (HF) hearts before (Day 0) and 42 d after (Day 42) myocardial infarction; D, E, Analysis of collagen content by means of hydroxyproline assay. Sham—sham‐operated group; HFni—non‐infarcted tissue; HFi—infarcted tissue. Data are presented as mean ± SEM, n = 7‐9 per group. *P < 0.05 vs Sham, ^#^ *P* < 0.05 vs HFni

Forty‐two days post‐MI, significant increase in both LVDd and LVDs, indicating the presence of left ventricular dilation, was found in failing hearts (Figure 3A‐B). Consequently, the FS index showed the significantly worsened LV systolic function in this group (Figure 3C).

The increased deposition of collagen in the non‐infarcted as well as in the infarcted area indicating increased interstitial collagen content and fibrotic healing supported echocardiographic findings on cardiac remodelling due to ongoing fibrosis (Figure 3D‐E).

### 3.2 Analysis of necroptotic signalling

**Figure 4:**
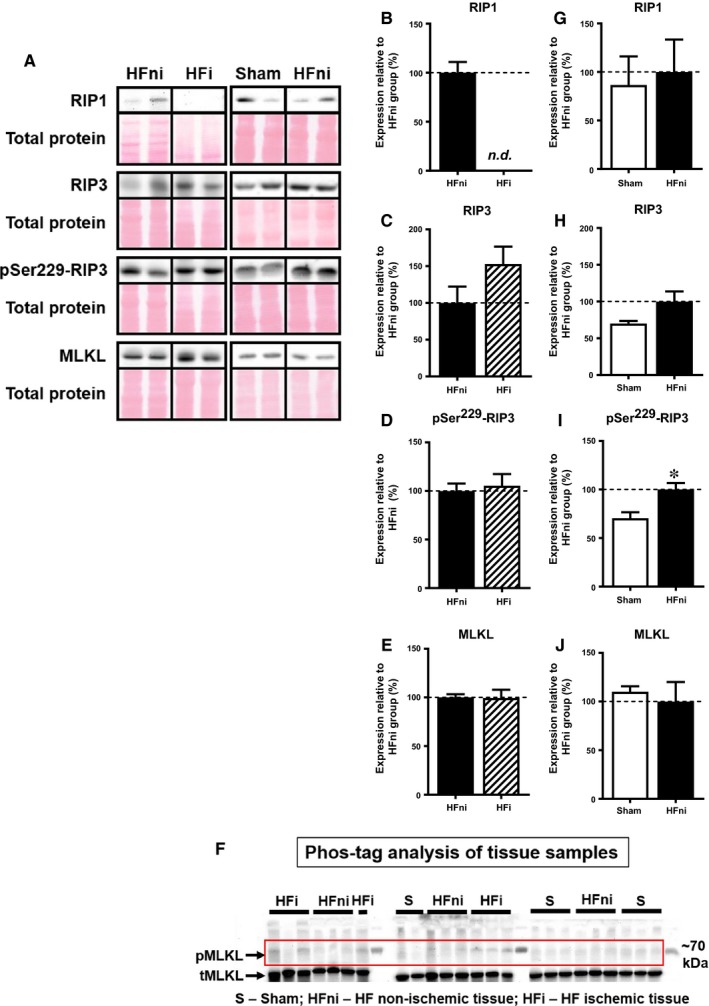
Analysis of necroptotic signalling in left ventricles lysates. A, Representative immunoblots and total protein staining; B‐E, G‐J, Immunoblot quantification of RIP1, RIP3, pSer229‐RIP3 and MLKL; F, Phos‐tag™ immunoblots. Sham—sham‐operated group; HFni—non‐infarcted tissue; HFi—infarcted tissue. Data are presented as mean ± SEM; n = 5‐9 per group; *P < 0.05 vs Sham; Abbreviation: n.d., non‐detectable

In the infarcted zone, RIP1 levels were not detected, and no significant difference in the levels of total RIP3 kinase between infarcted and non‐infarcted areas was found (Figure 4B‐C). Notably, the levels of pSer229‐RIP3, a direct upstream of MLKL activation, were unchanged in the infarcted zone when compared to the non‐infarcted area, however being upregulated when compared to sham group (Figure 4D,I). Expression of the terminal necroptotic pore‐forming protein MLKL was equal in both infarcted and non‐infarcted areas (Figure 4E). Of note, a phosphorylated form of MLKL, which is almost exclusively associated with MLKL oligomers formation in the cell membrane during necroptosis^10^, ^25^ was found to be heavily present in the infarcted zone (Figure 4F).

In the non‐infarcted zone, both RIP1 and RIP3 kinase expression was found to be unchanged compared with sham‐operated group (Figure 4G‐H). However, in this particular area of the failing hearts, the levels of pSer229‐RIP3 were significantly increased (Figure 4I). Nonetheless, while total MLKL protein expression did not increase, an upshifted band of MLKL representing its phosphorylated form was not consistently detected and found only in 1 out of 6 analysed samples in the non‐infarcted area of failing hearts, thereby suggesting no further mechanisms of necroptosis execution being activated in this zone (Figure 4F,J).

**Figure 5:**
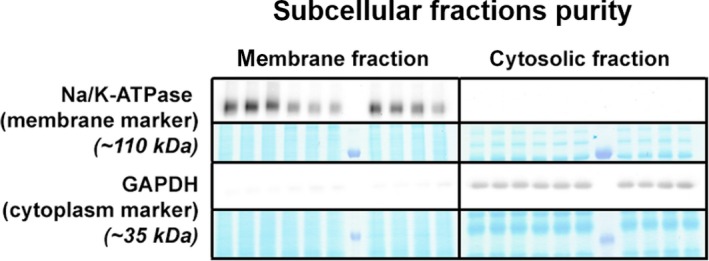
Assessment of subcellular fraction purity in membrane and cytosolic compartment of non‐infarcted and sham‐operated left ventricles. A, Representative immunoblots and protein expression of Na/K‐ATPase and GAPDH as markers of membrane and cytosolic fraction, respectively

**Figure 6:**
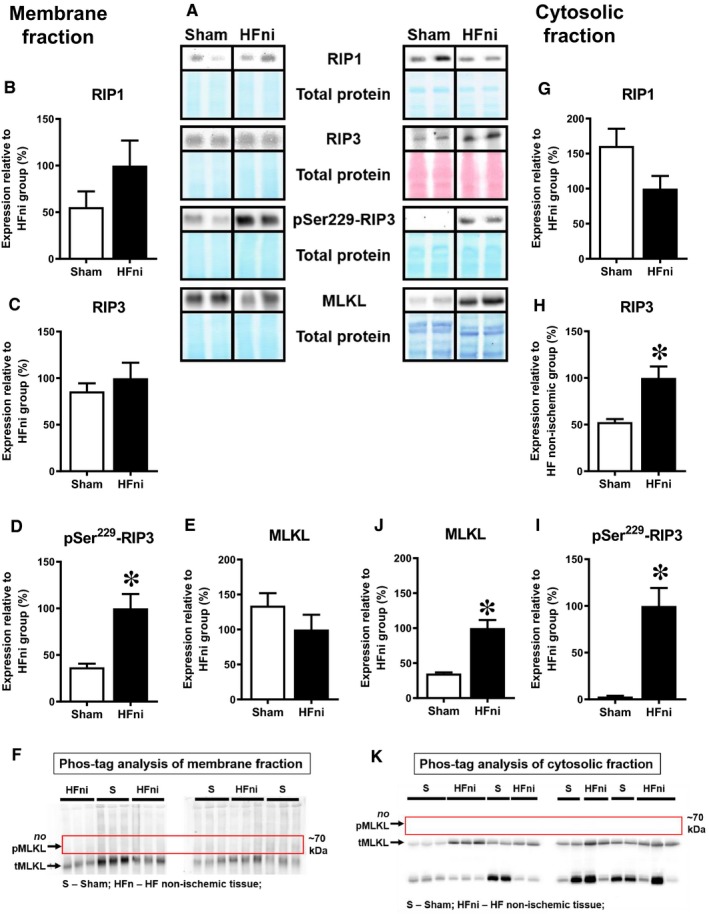
Analysis of necroptotic signalling in membrane and cytosolic fractions of non‐infarcted left ventricles. A, Representative immunoblots and total protein staining; B‐E, G‐J Quantification of RIP1, RIP3, pSer229‐RIP3 and MLKL in F, K, Phos‐tag™ immunoblots. Sham, sham‐operated group; HFni, non‐infarcted tissue; Data are presented as mean ± SEM; n = 6‐10 per group; *P < 0.05 vs Sham

To provide a more detailed insight into these findings and assess whether signalling of necroptosis is associated with protein relocation, we analysed the subcellular fractions in sham and non‐infarcted tissue. Fractions purity was verified by detection of Na/K‐ATPase and GAPDH as the markers of membrane and cytosolic compartments, respectively (Figure 5). The expression of all proteins (RIP1, RIP3 and MLKL) within the membrane, which is believed to play a major role in necroptosis execution, was similar to the expression detected in whole tissue lysates (Figure 6B‐E). On the other hand, an increased degree of RIP3 phosphorylation at Ser229 was observed in the non‐infarcted area compared with sham group (Figure 6D). In line with almost zero phosphorylation of MLKL found in the whole cell lysates of the non‐infarcted area, there was no evidence for this cytotoxic form of MLKL in the membrane, further confirming the absence of necroptosis execution in this particular area of failing hearts (Figure 6F). Analysis of cytoplasmic fraction of non‐infarcted HF tissue showed the unchanged levels of the total RIP1 kinase (Figure 6G). Contrarily, we observed an increased expression of both total and phosphorylated form of RIP3 (Figure 6H‐I). Although MLKL was significantly increased in the cytosolic fraction of the non‐infarcted tissue, no phosphorylated form was found in this area (Figure 6J‐K).

Altogether, expression and localization of the analysed proteins suggest necroptosis execution in the infarcted area while the findings in the non‐infarcted area of failing hearts indicate rather alterations being associated with pSer229‐RIP3 activation but not proceeding into MLKL‐dependent necroptosis.

### 3.3 Apoptotic markers expression

**Figure 7:**
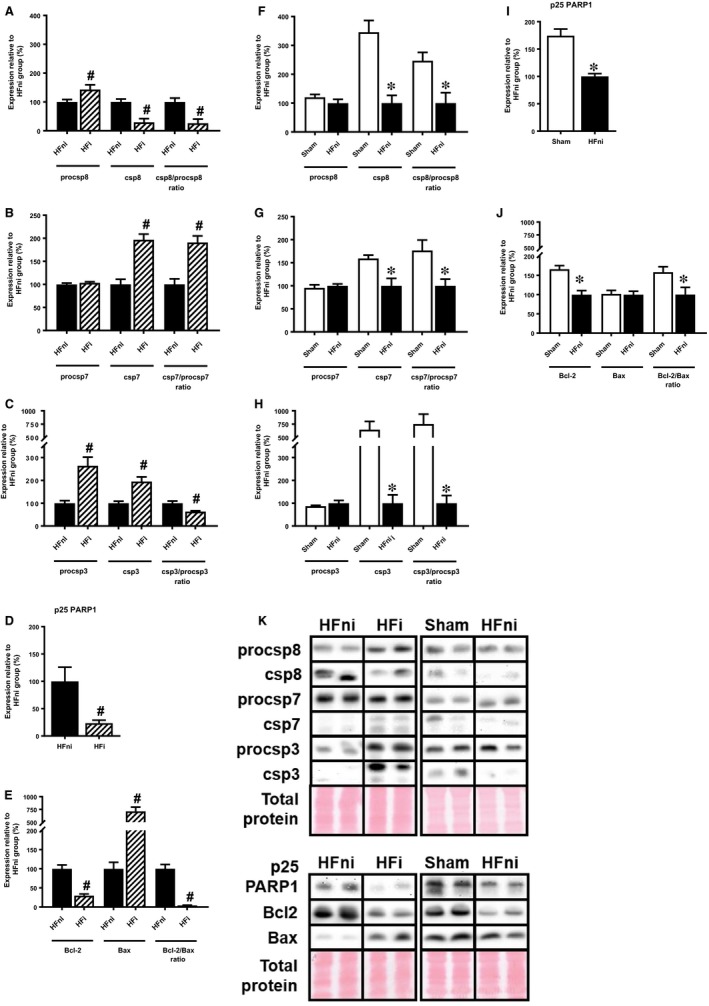
Analysis of apoptotic signalling in left ventricles lysates. A‐J, Quantification and K, representative immunoblots and total protein staining of procsp8, csp8, procsp7, csp7, procsp3, csp3, Bcl‐2, Bax and p25 PARP1 in sham‐operated group (Sham), non‐infarcted (HFni) and infarcted (HFi) tissue of failing hearts. Data are presented as mean ± SEM; n = 6‐9 per group; *P < 0.05 vs Sham; ^#^ *P* < 0.05 vs HFni

Because there is a caspase‐8‐dependent interlink between necroptotic and apoptotic signalling,^26^ we also analysed some well‐established markers of apoptotic cell death. In the infarcted zone, levels of active caspase‐8 were reduced despite its increased zymogene expression, suggesting a reduction in the caspase‐dependent signalling (Figure 7A). However, downstream caspase‐7 and caspase‐3 expression levels were increased relative to the non‐infarcted area (Figure 7B,C). Contrary to these changes, apoptotic PARP1 cleavage was the lowest in the non‐infarcted zone compared with other groups, and the Bcl‐2/Bax ratio showed a massive drop due to both a reduction in Bcl‐2 expression and an upregulation of Bax (Figure 7D,E).

The non‐infarcted zone was characterized by a different profile of these apoptotic markers compared with the infarcted one. Expression of almost all analysed pro‐apoptotic markers (active form of caspase‐8, executioner caspases −3 and −7 as well as specific apoptotic p25 fragment of PARP1) was reduced in the non‐infarcted zone compared with the sham‐operated group (Figure 7F‐I). Being significantly decreased, the Bcl‐2/Bax ratio was not in line with these findings (Figure 7J). Altogether, these data do not support the view that apoptotic cell death plays a significant role in post‐MI failing hearts.

### 3.4 Inflammatory response

**Figure 8:**
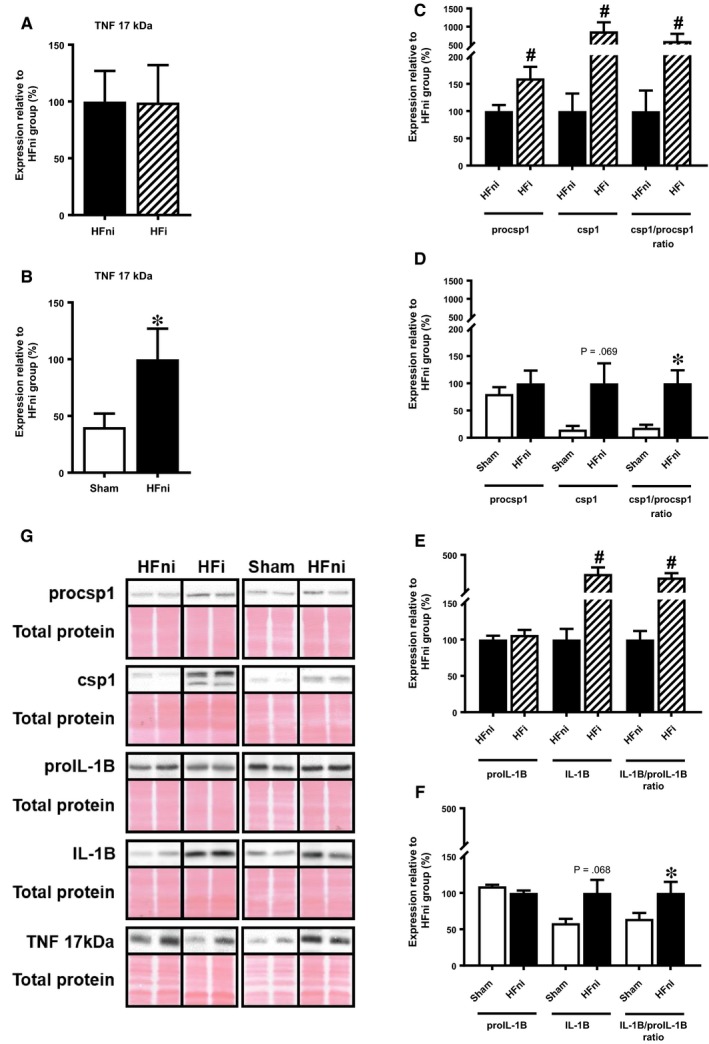
Analysis of pro‐inflammatory signalling in left ventricle lysates. A‐F, quantification and G, representative immunoblots and total protein staining of procsp1, csp1, proIL‐1β, IL‐1β and TNF in sham‐operated group (Sham), non‐infarcted (HFni) and infarcted (HFi) tissue of failing hearts. Data are presented as mean ± SEM; n = 6‐9 per group; *P < 0.05 vs Sham; ^#^ *P* < 0.05 vs HFni

Association between necroptosis and inflammation in the failing hearts was investigated by analysing expression of TNF, which has been proved to participate in both these processes,^27^, ^28^ and by NLRP3 inflammasome‐dependent activation of pro‐inflammatory signalling mediated by caspase‐1‐ IL‐1β axis.^16^, ^17^, ^29^ Expression of a cleaved 17 kDa TNF fragment was found to be similar in both infarcted and non‐infarcted tissue while being upregulated in comparison with sham group (Figure 8A,B). A relative processing of csp‐1 was significantly elevated in both zones of failing hearts, with zymogenic procsp‐1 being increased only in the infarcted tissue (Figure 8C,D). In line, the cleavage of proIL‐1β to its active IL‐1β analogue was significantly increased in both infarcted and non‐infarcted area, although proIL‐1β was decreased or unchanged in these areas, respectively (Figure 8E,F).

### 3.5 Oxidative stress

**Figure 9:**
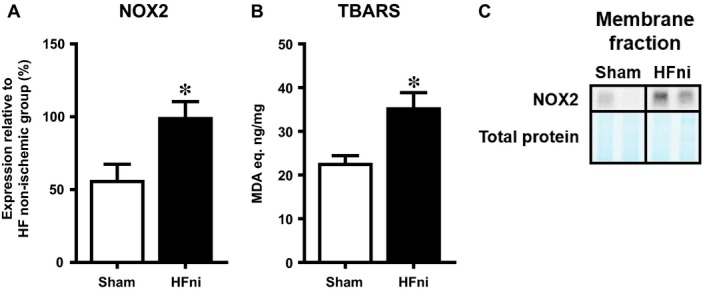
Analysis of oxidative stress in membrane fraction of left ventricles. A, Quantification of NOX2; B, analysis of membrane lipids peroxidation by means of TBARS assay and C, representative immunoblot and total protein staining of NOX2 in sham‐operated group (Sham) and non‐infarcted (HFni) tissue of failing hearts. Data are presented as mean ± SEM; n = 6‐9 per group; *P < 0.05 vs Sham

Because pro‐necroptotic RIP3 activation has been shown to be linked with increased ROS production,^3^ we also assessed oxidative stress associated with membrane lipid peroxidation and levels of NOX2, one of main pro‐oxidant membrane‐bound enzymes in the heart^30^ known to influence cardiac remodelling and chronic HF progression.^31^ In the membrane fraction of non‐infarcted tissue, where necroptosis is being executed in,^13^, ^14^ both these markers were higher than in the sham group (Figure 9A,B).

## 4 DISCUSSION

In this study, the canonical necroptotic signalling axis has been investigated for the first time in the infarcted and non‐infarcted area of rat hearts separately, and thereby, a novel mechanistic insight into its potential involvement in the pathogenesis of HF of ischaemic aetiology has been delineated. We have found that p‐MLKL, which has been shown to be related to necroptosis execution,^10^, ^25^ was almost exclusively present in the infarcted area of failing hearts where these cell loss associated alterations are likely to be an underlying cause of fibrotic healing. Non‐infarcted zone of LV of failing hearts with interstitial collagen deposition was without any changes in the expression of p‐MLKL on both cellular and subcellular levels. On the other hand, pSer229‐RIP3 serving as an upstream protein of MLKL was upregulated in both areas of failing hearts. Likewise, TNF and csp‐1‐IL‐1β axis, a pro‐inflammatory downstream pathway of RIP3,^29^, ^32^ were also found to be activated in both areas. It seems that in the infarcted area the activated RIP3 proceeds to MLKL signalling to further terminate in pro‐necroptotic events and inflammation while in the non‐infarcted area this activation of RIP3 can solely mediate pro‐inflammatory phenotype without necroptosis execution.

Excess cardiomyocyte damage occurring as a consequence of MI contributes to HF progression.^33^ In these cardiac pathologies, many conventional and less known cell death modalities have been identified,^33^ including necroptosis.^34^, ^35^ The canonical necroptotic signalling mostly relies on mutual phosphorylation of RIP1 and RIP3^11^, ^36^ with subsequent recruitment and phosphorylation of MLKL within this complex.^10^, ^37^ By such interaction, MLKL molecules form homo‐/heteromers which have been suggested to cause the rupture of plasma membrane due to the formation of ion specific/nonspecific pores.^10^, ^12^, ^13^, ^14^, ^25^ In this regard, the increased expression of proteins involved in necroptosis signalling (RIP1, RIP3, MLKL) and/or their activation due to phosphorylation (such as pSer227‐RIP3 or pSer357/Thr358‐MLKL) have been found in various models of ischaemia‐reperfusion injury and HF.^5^, ^6^, ^38^, ^39^, ^40^, ^41^ On the other hand, pharmacological inhibition of RIP1 or genetic depletion of RIP3 has been reported to reduce infarct size and suggested to limit adverse consequences of myocardial ischaemia.^2^, ^3^, ^6^, ^39^, ^41^, ^42^, ^43^ In addition to these findings shown in experiments on small animal models, we have also indicated pSer227‐RIP3 to be upregulated in LV of end‐stage human HF of various aetiologies, including ischaemic one.^5^ Likewise, phosphorylation of MLKL, which is uniquely associated with necroptosis execution,^10^ has been detected in human post‐MI failing hearts unlike in healthy ones.^5^ Nevertheless, it is completely unknown if and how this signalling differs in the infarcted and non‐infarcted area of hearts with such compromised function. This can be in particular important for the assessment of the progressive injury and the development of HF phenotypes.

In the present study, we have evaluated this approach by separately analysing these two particular LV areas of rat hearts 42 days post‐MI. In the heavily scarred infarcted area, we were unable to assess RIP1 expression, which was, however, similarly expressed in non‐infarcted tissue of failing hearts when compared to sham group. Because the infarcted tissue contained high levels of collagen, a decreased cellularity resulting in reduced RIP1 content could be an explanation for this difficulty. However, as other proteins of interest were easily detected in this area, we believe that specific RIP1‐associated cellular modifications could be rather a reason for such an absence of its signal. In support, we used various antibodies recognizing C‐ or N‐terminal of RIP1 (CST #3493; BD 610459), which were unable to detect any single signal for this protein, thereby arguing for most likely RIP1 degradation in the scared tissue. This finding is contrast with our previous study employing human post‐ischaemic failing hearts, in which, however, individual analysis of the ischaemic/infarcted versus non‐ischaemic/non‐infarcted area was not performed and highly fibrotic tissue was excluded for any molecular investigation.^5^ On the other hand, these data by themselves do not rule out active necroptosis in it because necroptosis can also occur independently of RIP1 in some conditions and activated RIP3 rather than RIP1 was found to be a key protein in necroptosis signal transduction.^44^ In our hands, there was no difference in the phosphorylated form of RIP3 between infarcted and non‐infarcted area; however, these levels were higher in comparison with sham‐operated group. On the other hand, the phosphorylation of MLKL, a downstream molecule of pSer229‐RIP3, which is believed to underlie cytotoxic effects of this pseudokinase due to the capability to assemble pore‐forming oligomers,^13^, ^14^, ^25^ was almost exclusively found in the infarcted zone. These findings have indicated ongoing necroptosis execution in the infarcted but not in the non‐infarcted area despite the comparably increased levels of TNF which is known to trigger necroptosis through TNFR1.^27^, ^36^ It should be noted, however, that Phos‐tag™ analysis used to detect p‐MLKL does not indicate which particular residue, Thr357, Ser358 or both, underwent phosphorylation, while in our previous study employing human end‐staged failing hearts of various aetiology, we found both these specific molecular necroptosis markers^10^ to be upregulated.^5^ Likewise, it can be mentioned that either of these phosphorylated MLKL residues is capable of MLKL‐mediated membrane permeabilization, ionic disbalance, oncosis and subsequent cell rupture.^25^, ^45^ As MLKL translocation to the plasma membrane has been detected in necroptosis positive tissues and it is considered to be another specific marker of necroptosis,^13^, ^14^, ^25^ we performed additional subcellular experiments to assess such necroptosis‐associated molecular events in non‐infarcted tissue of failing hearts and thereby to test if it can be possibly transduced also in this area. In the non‐infarcted tissue, the membrane content of total RIP1 and RIP3 was in line with their total cellular expression. On the other hand, in the cytosol, where interaction between RIP1 and RIP3 (mainly between their phosphorylated forms) and necrosome formation have been suggested to occur as a result of pro‐necroptotic stimuli,^11^ RIP1 expression remained unchanged. However, RIP3 and more importantly pSer229‐RIP3 levels were increased in HF, with latter closely mimicking its whole tissue content. Importantly, despite pSer229‐RIP3 being increased in whole cell lysates as well as in the particular subcellular fractions of the non‐infarcted samples of HF, no bands representing phosphorylated forms of MLKL were detected in any cellular/subcellular sample of this group. Thus, these findings suggest MLKL‐ and necroptosis‐independent role of enhanced RIP3 signalling in the non‐infarcted area of failing hearts. Interestingly, this may seem contradictory to conclusions from Luedde et al,^2^ who have shown that the overexpression of RIP3 alone in cardiomyocytes is a sufficient stimulus to promote necroptosis, It is noteworthy, however, to mention that in that study^2^ nor in any animal study employing various models of HF^3^, ^6^ MLKL, as a downstream molecule of RIP3, was not analysed. Such analysis of p‐MLKL, being present solely in the infarcted area unlike in non‐infarcted tissue, has been shown here for the first time. Although the authors of these studies^2^, ^3^, ^6^ have not commented not showing of such an important terminal protein in necroptotic signalling, we cannot rule out a possibility that RIP3‐mediated MLKL phosphorylation was actually present in their experimental settings and that it is not RIP3 alone which causes necroptotic cell death. Thus, such apparent disparities regarding RIP3‐MLKL signalling may be attributable to differences in experimental designs as well as different insights into necroptosis pathway.

As discussed above, the increased pSer229‐RIP3 did not materialize in MLKL phosphorylation in non‐infarcted area of failing hearts suggesting other mechanisms underlying the diffuse damage in this particular tissue. Thus, we have assessed RIP3 in the context of the RIP3‐NLRP3‐csp‐1‐IL‐1β axis promoting inflammation.^29^, ^32^ Such csp‐1‐mediated IL‐1β production has been found to stimulate chronic pro‐inflammatory tissue response and adverse ventricular remodeling after MI.^46^, ^47^, ^48^ In both LV zones of failing hearts, increased proIL‐1β cleavage has been demonstrated with the infarcted area being affected to a greater degree. By considering the fact that dying cells due to necroptosis exhibit plasma membrane rupture allowing the leak of intracellular content with resultant inflammation, it is difficult to distinguish whether such pro‐inflammatory environment in the infarcted tissue is an inevitable consequence of such cell‐destruction or if it is a separate, necroptosis‐independent process. In line with this idea, RIP3‐associated IL‐1β production by csp‐1 can be either MLKL‐dependent or independent and does not necessarily require necroptosis execution. It is very likely that which scenario occurs strongly depends on cellular conditions and pathological settings.^16^, ^17^, ^49^ Accordingly, under the conditions of this study, it can be postulated that the infarcted zone of HF is characterized by pro‐inflammatory TNF and IL‐1β elevation alongside signalling of necroptosis execution, while the alterations in non‐infarcted tissue with absenting p‐MLKL, and thereby exclusion of necroptosis, can rather be associated with solely inflammatory processes linking pSer229‐RIP3 and inflammatory cytokines. Supportive to these findings, it has been shown that TNF stimulation in cardiomyocytes, even in the presence of a pan‐caspase inhibitor, does not necessarily facilitate or potentiate necroptosis activation.^2^ Furthermore, considering the fact that TNF and IL‐1β can be involved in a process of endothelial‐to‐mesenchymal transition (EndMT),^50^, ^51^, ^52^, ^53^, ^54^ known to be a significant contributor to fibrosis 55, 56, 57 and inflammatory 51, 52, 53, 54 response, it can be hypothesized that in our study such an interconnection between RIP3‐NLRP3‐IL‐1β (TNF) and EndMT could contribute to the promotion of fibrotic remodelling. This phenomenon linking RIP3, EndMT and inflammation could be suggested as a novel mechanism of progressive tissue deterioration in HF which, however, requires further detailed investigation. Additionally, we have also assessed another harmful element interlinking necroptosis, inflammation and oxidative stress in the pathomechanisms of HF. In fact, RIP3 activation and IL‐1β production have been found to be in close relationship with ROS increase in several cell types including cardiomyocytes^3^, ^9^, ^18^ with NOX2 inhibition being protective against RIP3‐induced necroptosis in cardiomyocytes.^3^ In this study, the increased NOX2 expression and lipid peroxidation was found in the membrane compartment of non‐infarcted area with elevated pSer229‐RIP3. However, such increased membrane oxidative damage was not associated with p‐MLKL upregulation, rendering ROS‐necroptosis relationship insignificant, at least in this particular pathological setting.

When discussing other programmed cell death modalities with respect to the pathogenesis of failing hearts, apoptosis is of prime importance as it has been considered for decades to be one of the major cell death modes contributing to myocyte death.^58^ In our study, however, apoptosis signalling does not seem to be significantly involved in tissue damage either in infarcted or in non‐infarcted LV parts of failing hearts. These findings are in accordance with our results from a study employing end‐stage human failing hearts, and also with another work using a similar model of rat HF.^2^, ^5^ In contrast, some papers studying RIP3‐mediated necroptosis have reported increased apoptosis in HF.^3^, ^59^ This controversy only strengthens the fact that elevation of apoptosis as a major contributor to myocyte loss in HF progression is still a rather unresolved question requiring future clarification. To help clarify this issue, improvement of methods used to assess apoptosis (protein expression versus DNA laddering assessment and TUNEL assay) as well as considering different apoptosis rate between various heart‐residing cell types besides cardiomyocytes could provide some useful approaches for dealing with these discrepancies.^58^, ^60^, ^61^, ^62^ Interestingly, the apparent increase in executioner csp‐3 and csp‐7 expression in infarcted tissue did not result in increased PARP1 cleavage. Although we did not seek a detailed explanation for these findings, the previously described non‐apoptotic roles of caspases could provide a feasible link for future investigation.^63^, ^64^ Irrespective of this, it is likely that observations in apoptotic protein expression shown here do not suggest an exclusive role of apoptosis in the pathomechanisms of heart failure, at least at this stage.

## 5 CONCLUSIONS

**Figure 10:**
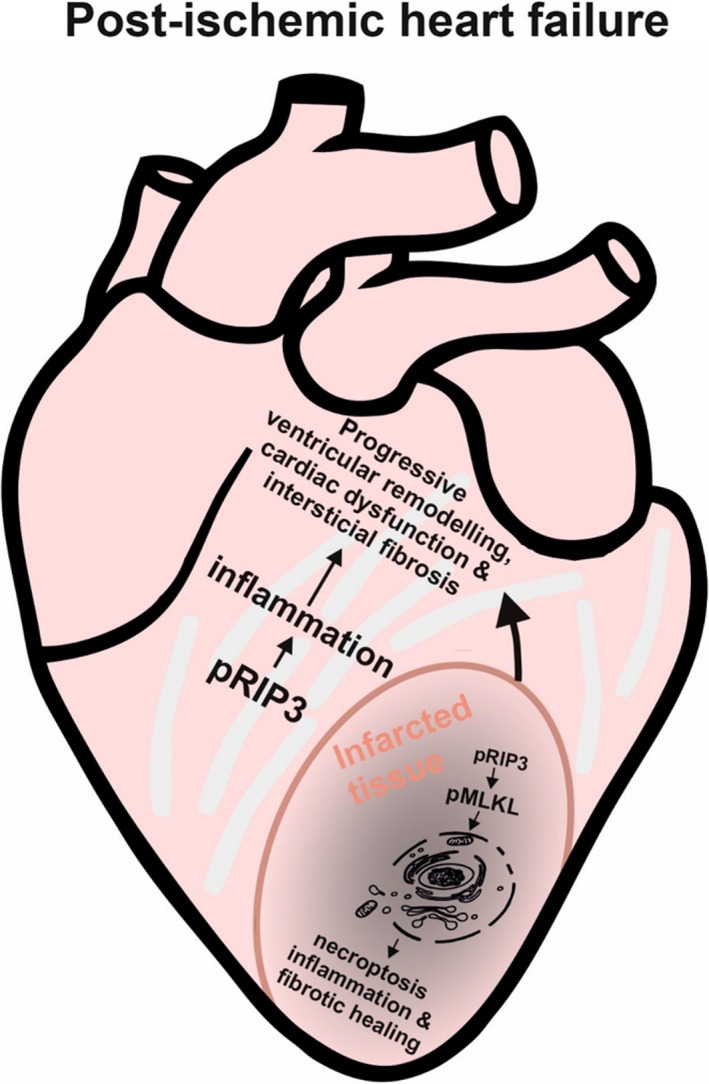
Proposed view of RIP3‐associated necroptosis and inflammation in infarcted and non‐infarcted area of failing heart

Taken together, although TNF and csp‐1‐IL‐1β‐associated inflammation has been present in both infarcted and non‐infarcted LV area of failing hearts, these particular myocardial parts are characterized by a different profile of cell death proteins. The canonical necroptotic signalling involving pSer229‐RIP3 and p‐MLKL was found in the infarcted area only. On the other hand, the non‐infarcted tissue has been found to exhibit pSer229‐RIP3 activation with possible resultant pro‐inflammatory rather than pro‐necroptotic signalling. Thus, it seems that this kinase might propagate different cellular responses, such as concurrent necroptosis and inflammation vs inflammation without necroptosis execution, in these particular parts of post‐MI failing hearts, demonstrating a novel insight on a proposed dual role of pSer229‐RIP3 in HF progression (Figure 10). Accordingly, these results have also highlighted a potential for selective pharmacological targeting of RIP3 to affect the two important processes contributing to progressive nature of HF.

### 5.1 Study limitations

Although we have provided novel findings about necroptosis signalling in the infarcted and non‐infarcted area of failing hearts and proposed dual role of pSer229‐RIP3 kinase, our study has some limitations. First, the study has mainly a descriptive character and pharmacological or genetic modulations targeting certain proteins in necroptosis and/or inflammation signalling would be desirable to confirm our original findings. Next, it would also be interesting to perform subcellular fractionation in the infarcted tissue which was, however, not subjected to this procedure because of technical issues (a small mass of scarred tissue not allowing to separate subcellular compartments). Lastly, additional biochemical or molecular experiments could contribute to the clarification of the proposed RIP3‐NLRP3‐mediated inflammation.

## CONFLICT OF INTEREST

The authors confirm that there are no conflicts of interest.

## AUTHOR CONTRIBUTIONS

ML, AS, JH, CH, VK, TR, JN performed the experiments, contributed to data analysis and participated on writing the manuscript; AA, FK developed the concept and coordinated all the work; AA, ML and FK wrote the manuscript. All authors read and approved the final version of the manuscript.

## Data Availability

The data that support the findings of this study are available from the corresponding author upon reasonable request.
